# Epidemiology and cardiovascular risk factors of aortic stenosis

**DOI:** 10.1186/1476-7120-4-27

**Published:** 2006-07-01

**Authors:** Pompilio Faggiano, Francesco Antonini-Canterin, Ferdinando Baldessin, Roberto Lorusso, Antonio D'Aloia, Livio Dei Cas

**Affiliations:** 1 Cattedra di Cardiologia, Università di Brescia e Divisione di Cardiologia, Ospedali Civili di Brescia – Italy; 2 Divisione di Cardiologia, Azienda Ospedaliera Santa Maria degli Angeli di Pordenone – Italy; 3 Divisione di Cardiochirurgia, Ospedali Civili di Brescia – Italy

## Abstract

The abnormalities of aortic valve morphology and function represent the most common cardiac-valve lesion particularly in elderly. The etiology of aortic stenosis is degenerative-calcific in the majority of patients. Many risk factors seems to be linked to the calcification and the stenosis of the aortic valve but they must be confirmed. In this review the etiology and the possible physiopathology of the aortic valve stenosis is discussed.

## Review

The abnormalities of aortic valve morphology and function represent the most common cardiac-valve lesion, with relevant implications both for medical and surgical treatment. Particularly, the aortic valve sclerosis (aortic valve thickening and calcification without pressure gradient) seem to affect about one fourth of adults over 65 years of age, while the aortic valve stenosis is present in 2–9% of general population over 65 years of age; an increased prevalence of both sclerosis and stenosis with aging (48% and 4% in those over 85 years) is observed. Furthermore, the number of the aortic valve procedures performed over the last 10 years is increasing if we consider the aortic valve replacement alone or combined with myocardial revascularization; mitral valve surgery seems to be constant in the same period. Also, the etiology of the pathologic process of the aortic valve is changing in the last years. Passik et al. analysed the valve characteristics in 646 patients with pure aortic stenosis who underwent valve replacement between 1981 and 1985 at Mayo Clinic. During the five years of the study, the relative frequency of the postinflammatory disease (i.e. post-rheumatic) decreased from 30% to 18% and the relative frequency of the bicuspid aortic valve changed from 37% to 33%; in contrast, the relative frequency of degenerative-calcific aortic stenosis (an "atherosclerotic" form of disease, see below) increased from 30% to 46%. These differences were striking in subjects older than 70 years [1].

Several studies have been published in the last years which clarify the epidemiology of valvular disease and, specifically, of aortic valve disease. In the The EuroHeart Failure survey programme, a survey on the quality of care among patients with heart failure in Europe, of 46.788 patients screened over a six-week period 11.327 (24%) patients were enrolled with a suspected or confirmed heart failure; a valve disease was the cause of heart failure in 29% of the cases, compared with coronary artery disease in 68% and idiopathic dilated cardiomyopathy in 6% [2]. In the Euro-Heart Survey of acute coronary syndromes, 489 out of 10207 (4.8%) patients with acute coronary syndromes enrolled had a significant valve disease. The more common abnormalities were ischemic mitral regurgitation and calcific aortic stenosis. The patients with valve disease were older, more likely females and with comorbidities such as diabetes and chronic renal failure; they more likely have had a prior CHF and LV dysfunction, cardiac ischemic event or revascularization. In-hospital and 30-day mortality of the patients with valve disease were significantly higher than those without (13.4% and 15.5% versus 6.4% and 7.7%, respectively) [3].

In the Euro Heart Survey on Valvular Heart Disease, aortic valve stenosis was the most common valve abnormality (33,9% and 46,6% in the overall group and surgical subgroup, respectively). The etiology of aortic stenosis was degenerative-calcific in the majority of patients (81,9%), while it was rheumatic in 11.2%, congenital in 5.6% and post-endocarditis in the remaining 1,3%. Among the 512 aortic stenosis patients who underwent valve replacement, 54,3% were elderly (more than 70 years), 80% had a preserved left ventricular systolic function (left ventricular ejection fraction >60%) and 85% had symptoms of heart failure (NYHA class II-IV) [4].

Other studies evaluated the prevalence of calcific aortic valve disease (sclerosis/stenosis) in the general population. In the Helsinki Ageing Study, among 577 apparently healthy subjects 75–86 years old some degree of aortic valve calcification (independently from pressure gradient) was observed in 53%; the prevalence of moderate to severe aortic stenosis (Doppler valve area <1.2 cm2) increased from 2.5% in the 75–76 years old group to 8.1% in the 85–86 years old group [5].

In the Cardiovascular Health Study, the Doppler echocardiographic examination performed in 5621 subjects older than 65 year without prevalent cardiovascular disease at entry identified an aortic sclerosis (valve thickening and iperreflectivity) in 29% of overall population and an aortic stenosis (valve abnormalities and instantaneous pressure gradient >25 mmHg) in 2% [6]. In hypertensive patients of INSIGHT study [7], some degree of aortic valve calcifications was present in 54% and in those of LIFE echo substudy (960 hypertensive patients between 55 and 80 years old) the frequency of the aortic sclerosis was 40.4% and that of aortic stenosis 1.6% at the ultrasound examination performed at admission; of particular interest, after a follow up of 4 years, the frequency increased to 63% and 4%, respectively [8].

Aortic valve sclerosis is commonly defined as a focal or diffuse thickening of the aortic cusps with calcific nodules generally at the base of leaflets and transvalvular velocity at Doppler still in the normal range (Vmax <2 m/s). Until few years ago, it was considered a physiologic process related to aging without clinical relevance. However, aortic valve sclerosis is not observed in about 50% of people over 80 years old. Furthermore, several experimental and clinical studies have showed that it could represent an active phenomenon, significantly related to risk factors for atherosclerosis and cardiovascular morbidity and morbility [9]. In the previously mentioned Cardiovascular Health Study, Stewart et Al. showed that clinical factors associated with aortic valve sclerosis and stenosis are similar to risk factors for atherosclerosis: age, male gender, height (inverse relation), smoking, history of hypertension, elevated serum levels of Lipoprotein (a), and LDL-cholesterol [6]. Furthermore, the rates of cardiovascular events during follow-up were significantly higher in subjects with compared to those without aortic valve sclerosis; the relative risk for cardiovascular mortality, acute myocardial infarction and congestive heart failure was 66%, 46% and 33% higher, respectively in subjects with aortic sclerosis. This excess of cardiovascular fatal and nonfatal events could not be exclusively attributed to complications, such as endocarditis and progression to severe aortic stenosis, of the few cases of significant aortic valve disease; thus, the hypothesis of an association between valve sclerosis and atherosclerotic coronary artery disease was formulated. In the Editorial comment to this landmark paper, Carabello proposed the use of aortic valve sclerosis as "*a window to the coronary arteries*?" [10].

William C. Roberts, the eminent cardiac pathologist and Editor in Chief of The American Journal of Cardiology, twenty years ago used the term "senile cardiac calcification syndrome" to indicate the simultaneous presence of calcific deposits in epicardial coronary arteries, in the mitral annular area, on the aortic valve cups and on the head of left ventricular papillary muscles. Roberts stated "... Thus, cardiac calcium is not good. It may narrow the coronary arteries, mitral valve orifice and aortic valve orifice and it may prevent either or both of these valvular orifices from closing completely. It is reasonable to believe that both mitral anular and aortic cuspal calcific deposits in the elderly have the same etiology as the coronary atherosclerotic plaques because the 3 are commonly present in the same heart and the predisposing factors of all 3 are the same." [11].

**Figure 1:**
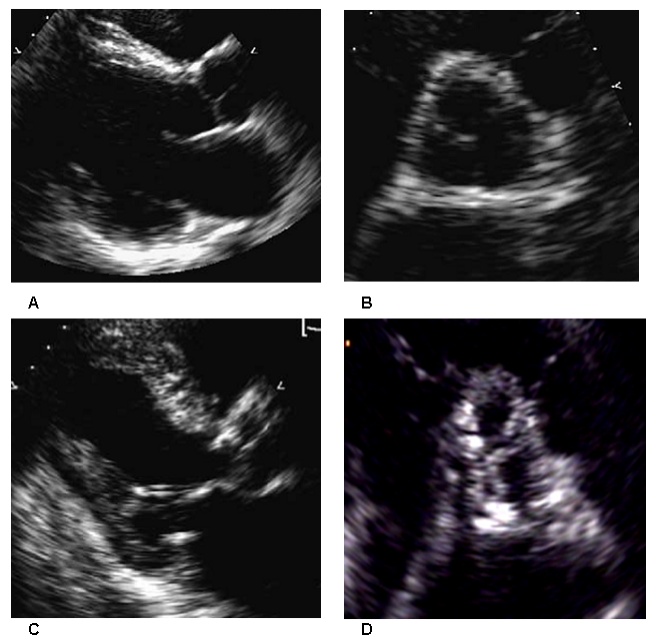
A – B – C – D Accordingly to the absence and presence cardiac calcifications identified, final score was calculated expressing the sum of all: it was in the range from 0 (no calcium visible) to 8 (severe calcification of aortic valve and mitral annulus, presence of calcium at the level of ascending aorta and papillary muscles). A – B No calcifications are visible at the level of aortic valve with a total calcium score of 0/8. C – D Calcifications are evident at multiple sites: mitral annulus, papillary muscles, aortic valve and ascending aortic wall with a total calcium score of 8/8.

Subsequent studies confirmed this interesting idea: the calcification of mitral annulus is associated with cardiovascular morbidity and mortality. In the Framingham Heart Study, 1197 subjects with an adequate echocardiogram were followed for 16 years; 14% had the calcification of mitral annulus. For each 1-mm increase in the calcification of mitral annulus size, the risk of incident cardiovascular diseases, cardiovascular mortality and total mortality, adjusted for relevant baseline risk factors, increased by ≈10% [12], (Figure 1) [13].

The progressive nature of aortic valve calcification has also been confirmed. Several studies documented the rate of progression of severity of valve stenosis over time (Table 1) [14]; according to these reports an average reduction of 0.1 cm2 in valve area per year of follow-up is generally observed; unfortunately, there is a large variability in the rate of progression among patients. Furthermore, many factors associated with a faster progression have been identified. Of interest, among these there are hemodynamic and valve-related variables, such as left ventricular function, bicuspid type of stenosis, initial severity of stenosis, but also, and specially, risk factors for atherosclerosis, such as age, smoking, hypertension, obesity and diabetes, lipid abnormalities, chronic renal failure and dialysis, and atherosclerotic disease itself, such as concomitant coronary artery disease.

**Table 1:** Findings associated to a faster progression of the aortic stenosis. The authors of the studies documented three subgroups of risk factors related to a faster progression: Patient-related; Hemodynamic-related; Valve-related.

**Patient-related**	
	Older age
	Smoking
	Hypertension
	Obesity/diabetes
	Lipid abnormalities
	Chronic renal failure
	Symptoms appearance or worsening
	Concomitant coronary artery disease
	
**Hemodynamic-related**	
	Left ventricular systolic dysfunction and/or low cardiac output
	Hemodynamic changes during exercise
	Dialysis
	
**Valve-related**	
	Bicuspid valve
	Degenerative aortic stenosis
	Valve calcification and regurgitation
	Mild-moderate stenosis at initial presentation

Patients on dialysis constitute a population at high risk for cardiovascular events and valvular calcification. In a group of patients on peritoneal dialysis the simultaneous presence of vascular (arterial) and cardiac valve calcifications is associated with an increased cardiovascular mortality risk of 73% compared to the absence of calcification [15]. Aortic valve calcification is frequent in end-stage renal failure and dialysis subjects ; among 155 hemodialysis patients referred to our echo laboratory in the last 12 months, some degree of aortic valve sclerosis was found in 66,5%, aortic valve stenosis in 17,5% and only 16% of subjects had a normal aortic valve (unpublished data). Furthermore, rate of progression of aortic stenosis is particularly fast in these patients [unpublished data]. Again, serum calcium-Phosphorus product, levels of Vitamin D and the well known cardiovascular risk factors represent favoring conditions.

Not only valve stenosis but even valve sclerosis is a progressive disease. Studies on rate of aortic valve sclerosis progression have been published recently. Faggiano et al. followed 400 subjects > 50 years old with aortic valve thickening/calcification and peak vel ≤ 2 m/s (valve sclerosis without stenosis) During a follow-up period of 44 ± 30 months, 131 patients (32.7%) developed some degree of aortic stenosis; furthermore, a pattern of rapid progression (rate of increase in peak aortic velocity 0.3 m/s/yr) was observed in 24/400 patients (6%); among 100 patients with a longer follow-up (> 5 years) a mild aortic stenosis appeared in 44%, a moderate in 14% and a severe one in 8% [16].

In the LIFE study, the risk for developing aortic valve stenosis was greater in patients with aortic valve sclerosis compared with those with normal valve at baseline, after 1 year (2.8% versus 0.4%, p < 0.001) and after 4 years (6.9% versus 0.9%, p < 0.001) of antihypertensive treatment. Patients with abnormal compared to those with normal aortic valves throughout the study had a greater incidence of composite end points (16.8% versus 9.3%, p 0.05) confirming previous studies. The prevalence of aortic valve sclerosis and mild aortic valve stenosis increased continuously in this elderly, high-risk hypertensive population, and this progression was prevented by neither losartan- nor atenolol-based treatment [8]. On the other hand, Antonini-Canterin et Al. recently reported the results of a retrospective study showing that long-term therapy with lipid-lowering drugs, such as statin, was able to slow the rate of progression of aortic valve sclerosis [17].

## Conclusion

Abnormalities of the aortic valve, without (aortic sclerosis) or with obstruction to left ventricular outflow (aortic stenosis) are particularly frequent in the general population, are associated to a high incidence of cardiovascular events, are related to risk factors for atherosclerosis and share similarities with atherosclerotic plaques (see other sections of this supplements). The knowledge of pathogenetic mechanisms, rate of progression over time and effects of medical treatment can guide our approach to this common disease.

## Competing interests

The author(s) declare that they have no competing interests.

## Authors' contributions

PF designed the study

FAC participated in the design of the study

FB helped to draft the manuscript

RL helped to draft the manuscript

ADA helped to draft the manuscript

LDC helped to draft the manuscript

All authors read and approved the final manuscript
